# D-MAINS: A Deep-Learning Model for the Label-Free Detection of Mitosis, Apoptosis, Interphase, Necrosis, and Senescence in Cancer Cells

**DOI:** 10.3390/cells13121004

**Published:** 2024-06-08

**Authors:** Sarah He, Muhammed Sillah, Aidan R. Cole, Apoorva Uboveja, Katherine M. Aird, Yu-Chih Chen, Yi-Nan Gong

**Affiliations:** 1 Department of Biological Sciences, Carnegie Mellon University, 5000 Forbes Avenue, Pittsburgh, PA 15213, USA; sxh@alumni.cmu.edu; 2 Hillman Cancer Center, UPMC, 5115 Center Avenue, Pittsburgh, PA 15232, USA; mus21@pitt.edu (M.S.); apu7@pitt.edu (A.U.);; 3 Department of Immunology, University of Pittsburgh School of Medicine, 3420 Forbes Avenue, Pittsburgh, PA 15260, USA; 4 Department of Pharmacology & Chemical Biology, University of Pittsburgh School of Medicine, 3420 Forbes Avenue, Pittsburgh, PA 15260, USA; 5 Department of Computational and Systems Biology, University of Pittsburgh School of Medicine, 3420 Forbes Avenue, Pittsburgh, PA 15260, USA; 6 Department of Bioengineering, Swanson School of Engineering, University of Pittsburgh, 3700 O’Hara Street, Pittsburgh, PA 15260, USA; 7 CMU-Pitt Ph.D. Program in Computational Biology, University of Pittsburgh, 3420 Forbes Avenue, Pittsburgh, PA 15260, USA

**Keywords:** cell death, senescence, label free, mitosis, interphase, machine learning, image processing

## Abstract

Background: Identifying cells engaged in fundamental cellular processes, such as proliferation or living/death statuses, is pivotal across numerous research fields. However, prevailing methods relying on molecular biomarkers are constrained by high costs, limited specificity, protracted sample preparation, and reliance on fluorescence imaging. Methods: Based on cellular morphology in phase contrast images, we developed a deep-learning model named Detector of Mitosis, Apoptosis, Interphase, Necrosis, and Senescence (D-MAINS). Results: D-MAINS utilizes machine learning and image processing techniques, enabling swift and label-free categorization of cell death, division, and senescence at a single-cell resolution. Impressively, D-MAINS achieved an accuracy of 96.4 ± 0.5% and was validated with established molecular biomarkers. D-MAINS underwent rigorous testing under varied conditions not initially present in the training dataset. It demonstrated proficiency across diverse scenarios, encompassing additional cell lines, drug treatments, and distinct microscopes with different objective lenses and magnifications, affirming the robustness and adaptability of D-MAINS across multiple experimental setups. Conclusions: D-MAINS is an example showcasing the feasibility of a low-cost, rapid, and label-free methodology for distinguishing various cellular states. Its versatility makes it a promising tool applicable across a broad spectrum of biomedical research contexts, particularly in cell death and oncology studies.

## 1. Introduction

It is well known that in the field of cancer biology, senescence, cell division via mitosis, and cell death play a significant role. Cancer cells undergo these different stages under certain therapeutic pressures, which have their own unique consequences and effects on the tumor microenvironment. More specifically, senescence is commonly induced by chemotherapies and can often lead to undesirable effects, such as promoting pro-tumor effects in the tumor microenvironment [1,2]. Cellular senescence can also be more immunogenic as it upregulates MHC-I and promotes dendritic cell—CD8 T cell-mediated anti-tumor immunity [3]. Mitosis and cell death are usually precisely regulated, but they are dysregulated by oncogenic signals in cancer cells; this results in uncontrolled proliferation and unwanted survival, promoting a pro-tumor environment. Decoding these pathways has led to advances in understanding tumor biology pathways and creating therapeutic approaches. Many efforts are currently underway to develop reagents and therapeutics that regulate senescence and cell death [4,5]. Therefore, it is essential to distinguish cell statuses in senescence, mitosis, and cell death in a timely, accurate manner.

Cellular morphology can be one of the most straightforward methods to distinguish cell states in senescence, mitosis, and cell death. The cellular morphology for these cells is standard for most adherent cancer cell lines. Senescence is categorized by abnormally increased cell size and flattened features [6]. Cell death can be split into at least two categories including apoptosis and necrosis. Apoptosis is a non-lytic form of cell death, categorized by plasma membrane (PM) blebbing and cell shrinkage. Necrosis is categorized by PM breakdown, cell swelling, and lysis [7]. There also exists a form of cell death called autophagic cell death; however, the molecular mechanisms and morphological features are less well-defined compared with apoptosis or necrosis. As such, D-MAINS is not developed to probe autophagic cell death. These morphological distinctions can be easily detected by experienced researchers in the field by visual inspection under bright field or phase contrast microscopy. However, visual inspection can be subjective to the researcher’s experience and more qualitative when counting thousands of cells.

In addition to visual inspection, other cell death probing methods involve biomarker stains and assays. These methods are often costly, inefficient, and qualitative. Stains such as Propidium Iodide (PI) or SYTOX dyes can distinguish between apoptosis and necrosis for cell death. More specifically, SYTOX Green (or PI) is used because it can stain the nucleic acids of cells with compromised plasma membranes, which only applies to necrotic cells. Annexin V was also used for apoptotic labeling, but it is now known that it also stains necroptotic cells [8]. Mitosis can be characterized by DAPI staining of the nucleus; chromosome condensation is a hallmark feature of a cell undergoing mitosis [9]. For senescence, the most common assay for detection is the senescence-associated β-galactosidase (SA-β-gal) assay. This assay is pH sensitive, requires costly reagents, takes considerable time to perform, and is mainly qualitative rather than quantitative [10,11,12]. The cells must also be fixed for the SA-β-gal assay; thus, live imaging for senescent cell dynamics is almost impossible. Overall, staining not only takes additional time and cost to perform, but it also takes up channels that can be used for biomarker detection. For example, suppose a fluorescent fluorescein isothiocyanate (FITC)-based dye is used to detect senescence. In this case, the GFP channel cannot be used to detect other biomarkers that might be important and not easily detectable by morphology. Thus, it is important to develop a label-free method in order to overcome these challenges.

Recent advancements in machine learning and other technologies have led to new automated, high-throughput techniques [13]. These techniques are beneficial not only because of their ability to have high throughput but also because they are label-free and can detect live or continuous cell statuses [14,15,16,17]. Within D-MAINS, we use convolutional neural networks (CNNs). A convolutional neural network (CNN) is especially effective at high-throughput image classification [18,19]. A CNN is a supervised learning algorithm that can take images with preset categories and spot which features make the images part of their specific category. It works by separating the images into multiple layers and detecting patterns from each layer. CNNs are designed to automatically and adaptively learn spatial hierarchies of features from input images, which makes them very efficient and ideal for tasks like image classification and object detection. Compared with traditional image detection methods, CNNs achieve higher accuracy and are more flexible for complex image detection. While CNNs require a large dataset, this is minor compared with the accuracy they can achieve and the fact that training is only performed once to use the network indefinitely. After training, the classification based on the trained dataset is performed very quickly in a matter of seconds. Currently, CNNs are the most advanced method for complex and accurate image analysis. With this method, we are able to assess new images using the information obtained from the training images.

Previously, a similar machine learning method was used to detect living/dead cell states in multiple cell lines in our lab [8]. Here, we report an advanced model that can recognize mitotic and senescent cells, in addition to apoptotic and necrotic cells. This novel algorithm allows for an accurate and efficient label-free method to detect mitosis, apoptosis, interphase, necrosis, and senescence; thus, it is named D-MAINS.

## 2. Materials and Methods

This section describes how we built the D-MAINS algorithm in detail.

### 2.1. Cell Culture and Induction of Senescence and Cell Death

HeLa cells were obtained from the ATCC and were cultured in DMEM with 10% FBS, 2 mM L-glutamine (GIBCO, Grand Island, NY, USA), 200 U/mL penicillin−streptomycin (GIBCO, Grand Island, NY, USA), and 50 μg/mL Plasmocin (Invivogen, San Diego, CA, USA) maintained at 37 °C with 5% *v*/*v* CO_2_ in a humidified incubator. Ovcar8 cells were a gift from Dr. Benjamin Bitler (University of Colorado) and were cultured in RPMI 1640 (Fisher Scientific, Hampton, NH, USA) supplemented with 5% Fetal Bovine Serum (BioWest, Bradenton, FL, USA) and 1% penicillin/streptomycin (Fisher Scientific). NIH3T3 cells (ATCC, Manassas, VA, USA) were cultured in DMEM with 10% FBS, 2 mM L-glutamine (GIBCO), 200 U/mL penicillin–streptomycin (GIBCO) and 50 μg/mL Plasmocin (Invivogen) maintained at 37 °C with 5% *v*/*v* CO_2_ in a humidified incubator. All cell lines were tested regularly for mycoplasma by a PCR assay using tissue culture supernatant.

For HeLa cells, 8 μM of etoposide (Sigma, Burlington, MA, USA) was added directly to the culture media in a 6-well plate for a total of 6 days before imaging to induce senescence. Then, 20 ng/mL TNF-α (peprotech) + 20 μg/mL cycloheximide (TNF+CHX) was used for 3 h to induce cell death in the HeLa cells. The TNF+CHX condition induces apoptosis following secondary necrosis (e.g., GSDME-mediated pyroptosis) [19].

For Ovcar8 cells, 1 µM cisplatin (Selleck Chemicals, Houston, TX, USA) was used to induce senescence and cell death. Ovcar8 cells were treated with cisplatin for 48 h, after which the cisplatin-treated cells were washed with PBS and cultured in fresh media for another 72 h.

For NIH3T3 cells, 50 µM etoposide (Sigma) was added directly to the culture media in a 6-well plate for 48 h before imaging to induce senescence and cell death.

Determination of etoposide and cisplatin concentrations was performed via titration to maximize senescent cells for imaging. The starting concentrations of etoposide and cisplatin were defined by previous studies [20,21].

### 2.2. β-Galactosidase Assay

SA-β-Gal staining was performed as previously described [22]. Ovcar8 cells were fixed in 2% formaldehyde/0.2% glutaraldehyde (Fisher Scientific; cat#BP531-25, #01909-100, respectively) in PBS (5 min) and stained (40 mM Na_2_HPO_4_ (Sigma cat#S9763-1KG), 150 mM NaCl (VWR, Radnor, PA, USA cat#97061-266), 2 mM MgCl_2_ (VWR cat#0288), 5 mM K_3_Fe(CN)6 (Arcos Organics, Geel, Antwerpen, Belgium, cat#223111000), 5 mM K_4_Fe(CN)_6_ (Alfa Aesar, Ward Hill, MA, USA cat#A15736-22), and 1 mg/mL X-gal (Sigma cat#B4252)) overnight at 37 °C in a non-CO_2_ incubator. The cells were imaged and analyzed 1–2 days after β-galactosidase staining.

### 2.3. Hoechst 33342, Annexin V and SYTOX Green Staining

HeLa cells were imaged after 5 min for Hoechst 33342 staining following the manual (Invitrogen, Waltham, MA, USA). For Annexin V and SYTOX Green staining, Alexa fluor 647-labeled Annexin V (1:200, Invitrogen) and SYTOX Green (Invitrogen, 50 nM) were added at the same time when HeLa cells were stimulated by TNF+CHX.

### 2.4. Image Acquisition

To train the D-MAINS model, the model’s training data comprised grayscale phase contrast images captured using the Lionheart FX automated microscope (Agilent Technologies, Santa Clara, CA, USA) equipped with Gen5 software (Version 3.05, Agilent Technologies, Santa Clara, CA, USA, https://www.agilent.com/en/product/cell-analysis/cell-imaging-microscopy/cell-imaging-microscopy-software/biotek-gen5-software-for-imaging-microscopy-1623226#literature (accessed on 3 June 2024)) at 20× magnification. During model verification, images were obtained using Lionheart FX Gen 5 and Nikon Ti2E (Nikon USA, Melville, NY, USA) microscopes: Lionheart FX images were captured at 20× magnification (phase contrast), while Nikon Ti2E images were taken at 10× magnification (bright field). To minimize potential phototoxic effects on the cells, phase contrast, and bright field imaging were conducted with an exposure time of less than 100 ms. Color bright field (Lionheart FX microscope) imaging was utilized for detecting β-gal staining, the DAPI filter facilitated Hoechst staining detection, and GFP and Cy5 filters facilitated SYTOX Green and Annexin V-Alexa Fluor 647 staining, respectively. All fluorescent staining images were captured by the Lionheart FX microscope.

### 2.5. Image Processing

**Figure 1:**
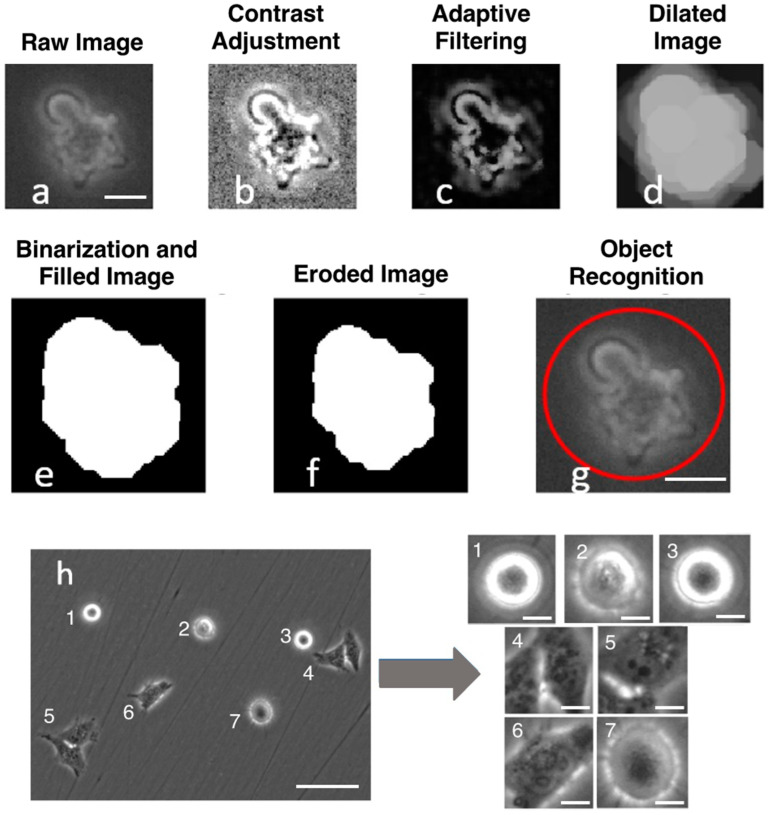
Cell cropping process. (**a**–**f**) Single-cell cropping process. (**a**) Unaltered pre-cropped raw image from the microscope. Scale bar = 10 µm, (**b**) Contrast adjustment for an improved binarization outcome. (**c**) Adaptive filtering through a 5 × 5 window. (**d**) Dilated image to fill in border irregularities. (**e**) Binarized image with inside filled in. (**f**) Eroded image to minimize multiple cell detection, areas under 2000 pixels are removed. (**g**) Cell cropping based on the centroid. Scale bar = 10 µm. The red circle signifies that the object inside (the cell) is detected by the cell cropping process. (**h**) An example of a raw 20× microscopy image from the BioTek Lionheart FX microscope using previous methods to detect and crop cells into 100 × 100 pixels. Scale bar = 100 µm for uncropped images, 10 µm for cropped images.

The primary aim of image processing was to standardize image sizes containing individual cells, which was achieved through MATLAB R2023a. Image processing involved contrast adjustment (Figure 1), followed by adaptive filtering using a 5 × 5 filter to minimize additional noise from image adjustments. Subsequent dilation expanded cell borders for complete connectivity. Binarization facilitated cell border detection, with the filled-in borders revealing the entire cell structure. Erosion techniques reduced detection areas to prevent multiple cell detections. Elimination of small objects under 30 × 30 pixels was performed because of their probable debris nature. Finally, a 100 × 100-pixel image was cropped from the detected cell’s centroid and subsequently saved for model testing and training.

**Figure 2:**
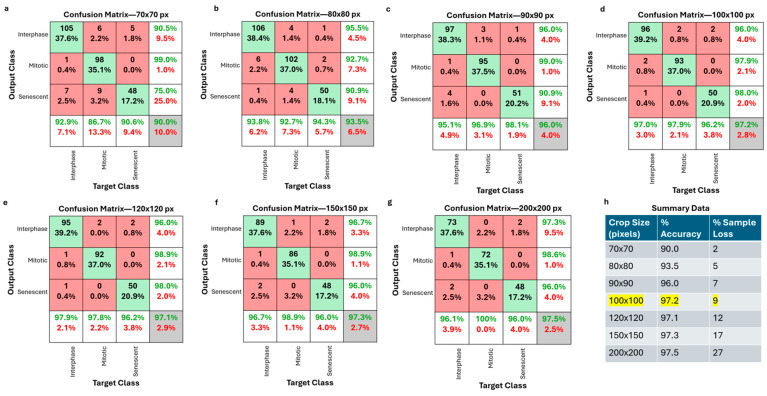
Optimization of cell cropping size. (**a**–**g**) Representative confusion matrices of crop sizes varying from 70 × 70 pixels to 200 × 200 pixels. (**h**) Summary table showing % accuracy and % sample loss for the different crop sizes. Highlighted row is the crop size used for D-MAINS.

100 × 100-pixel images were used with careful and rigorous empirical evaluations. Smaller windows, e.g., 90 × 90 pixels, risk decreased accuracy by potentially losing crucial parts of cells (Figure 2). Conversely, larger windows, e.g., 150 × 150 pixels, may discard more cells because of the presence of multiple cells in an image as well as cells on the border being excluded because of the inability to crop 150 × 150 pixels from the centroid without overstepping the bounds of the image (Figure 2). As such, the selected 100 × 100-pixel cropping window was determined empirically. Although 100 × 100 pixels may truncate cell parts (Figure 1h), the precision of cropped images sufficiently reveals crucial cell features within the confines of 100 × 100 pixels, as supported by our high prediction accuracy, which will be shown later. Moreover, centering crops at cell centroids was used to eliminate the impact of cell orientation on the cropping region. From cell cropping, part of the cell boundary may be lost; however, based on testing crop sizes in Figure 2, the whole cell boundary is not needed for the high accuracy generated by the deep learning model.

This standardized process was consistent across various microscopes and cell types. To ensure compatibility, we resized 10× images to 20× before testing, all saved as .tif 16-bit files.

### 2.6. Database of Images for Training

**Figure 3:**
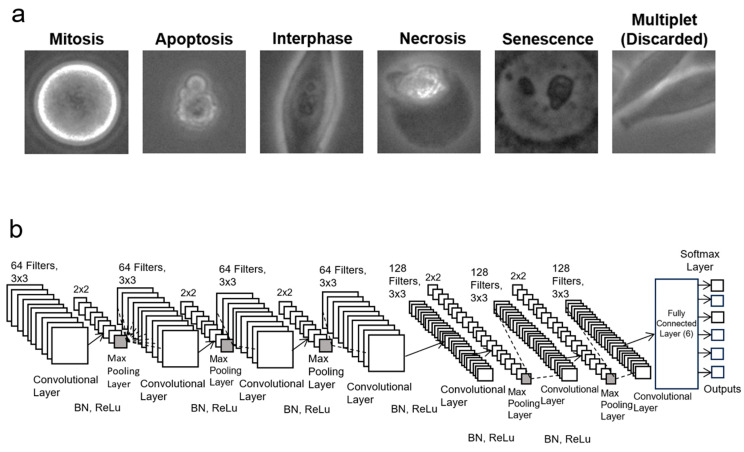
Establishing the D-MAINS detection model. (**a**) Representative images of cell state categories (“mitosis”, “apoptosis”, “necrosis”, “interphase”, “senescence”, and “multiplet” (discarded cells)) that were used in the training datasets. Scale bar = 10 µm. (**b**) The convolutional neural network (CNN) structure is characterized by seven convolutional layers.

HeLa cells were used for training the D-MAINS model. Representative images of “senescence”, “apoptosis”, “necrosis”, “interphase”, “discarded”, and “mitosis” were selected based on visual inspection and later confirmed by molecular markers. “Senescence”, “apoptosis”, and “necrosis” morphologies were defined by the literature [6,7,9]. Senescence is marked by flat and dark cells. We induced HeLa cells undergoing senescence by etoposide for 6 days. Apoptosis was characterized by apoptotic PM blebbing and cell shrinking, while necrosis was characterized by cell swelling. We saw both types of cell death in TNF+CHX conditions after a 3 h treatment. Although TNF+CHX is a classic apoptosis inducer, apoptotic HeLa cells can undergo secondary necrosis (most likely GSDME-mediated pyroptosis) [23]. Interphase was characterized by a slightly oblong shape with clear boundaries [4,5,6]. Mitosis was defined by a round, circular shape with a bright border. For mitosis, we found ~10% mitotic HeLa cells in normal cell culture conditions, so mitosis was not synthetically induced. The discarded category included multiplet cells that touch and contain multiple cells in the cropped image. Debris was filtered out during the cell cropping stage. Examples of these cropped images are presented in Figure 3a. Each category comprised approximately 500 images in the training database, totaling over 3000 images for the entire dataset. The training dataset images were manually captured on the BioTek Lionheart FX Gen 5 microscope and labeled into their respective categories before training.

### 2.7. Convolutional Neural Network Architecture

The convolutional neural network (CNN) architecture employed in this study is a sophisticated framework meticulously designed to extract intricate features from the input images. Comprising seven convolutional layers to capture the nuances present in the visual data, the network employs a range of filters spanning from 64 to 128. These filters play a pivotal role in discerning and identifying intricate patterns embedded within the images, thereby enabling the network to comprehend and interpret complex visual information effectively. To enhance the robustness and efficiency of the model, batch normalization techniques are incorporated, ensuring stable and consistent training dynamics across the layers. Furthermore, the inclusion of rectifier function layers (ReLu) serves to standardize the inputs while introducing nonlinearity into the dataset, thereby facilitating enhanced optimization and model efficiency.

During the initial stages of feature extraction, 64 filters are strategically employed in the convolutional layers to detect larger patterns inherent in the images. As the network progresses through subsequent layers, this number is augmented to 128 filters, enabling the detection of finer details and subtler nuances present within the images. To further augment the network’s capability in recognizing and generalizing patterns, max-pooling layers are integrated, operating over a 2 by 2 matrix with a stride size of 2. This pooling mechanism ensures that the network captures the most prominent features by selecting the maximum value from each region, thereby promoting translational invariance within the dataset.

The culmination of these convolutional and pooling layers is manifested in a fully connected layer, which serves as the cumulative layer for synthesizing the extracted features and facilitating comprehensive pattern recognition. This fully connected layer is important in calculating a SoftMax layer, which in turn computes the probabilities associated with each class, leveraging the intricate patterns discerned by the preceding convolutional layers. Notably, the architectural blueprint of the CNN employed in this study resembles the LANCE model, a precursor known for its efficacy in detecting cell death in suspension cells [8]. To tailor the model specifically for the complexities inherent in adherent cells and diverse cell states, a further augmentation is introduced in the form of an additional convolutional layer housing 64 filters compared with the LANCE model. This augmentation is necessary for the D-MAINS framework, given the expanded spectrum of categories necessitating a heightened capacity for pattern detection and classification. Consequently, the incorporation of this supplementary convolutional layer stands as an enhancement that elevates the accuracy and efficacy of the model in discerning the crucial patterns inherent in the dataset for cell classification.

### 2.8. Convolutional Neural Network Training and Validation

**Figure 4:**
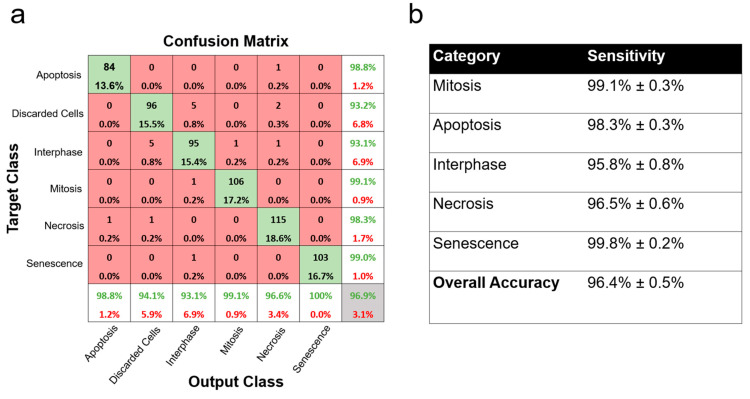
D-MAINS accuracy and sensitivity. (**a**) Confusion matrix of the training dataset after one trial. (**b**) Table of accuracy and sensitivities of each cell status on HeLa cells for the training and validation dataset.

By using the MATLAB 2023a deep learning toolbox, we established and trained a CNN model to detect six categories as follows: “senescence”, “apoptosis”, “necrosis”, “interphase”, “discarded”, and “mitosis” (Figure 3). Overall, 80% of the dataset was used for training and 20% was used to test the validation accuracy of the model, which is a common practice for machine learning based on the Pareto Principle [24]. Additionally, multiple training options were applied within the MATLAB training options. Adaptive Moment Estimation (Adam) was used to optimize the CNN [25]. Within Adam, the initial learning rate was changed to 0.01 in order to increase the efficiency of the training and minimize divergence. The maximum number of epochs was chosen to be 20, which means the training concluded after 20 rounds of using the CNN model across the entire training dataset. The learning rate was set to drop every 10 epochs in a piecewise manner. After each epoch, the training dataset was randomly shuffled in order to provide variance with each pass. The training dataset was split into mini-batches of 128. The validation frequency was set to 50, meaning validation was performed at every 50 mini-batch of data. “Verbose” was applied to visualize the training data in a separate window while the training was performed. In order to prevent overfitting, an L2 regularization was applied at 0.005. Additionally, a validation patience was applied to terminate the training if the validation performance did not improve after 5 epochs. The accuracy and sensitivity of D-MAINS cell state classification were measured using the following equations using the test dataset (Figure 4):

$$Accuracy\;=\;\frac{\#\;correctly\;categorized\;images}{\#\;total\;images\;in\;the\;testing\;dataset}$$

$$Sensitivity\;=\;\frac{\#\;correctly\;categorized\;images\;in\;specific\;category}{\#\;images\;in\;specific\;category}$$

Images identified by the cell cropping model were classified with the CNN training model. Objects in the “discarded cells” category included multiplet cells and were excluded from the calculations. Cells on the edges of each image were also excluded from the calculations. “Mitosis”, “apoptosis”, “interphase”, “necrosis”, and “senescence” events were calculated subsequently (Figure 4).

### 2.9. D-MAINS Algorithm Code Accessibility

The D-MAINS algorithm was created in MATLAB R2023a, and the cell cropping and training network code was uploaded to GitHub as a public source code [https://github.com/cakekio/D-MAINS (accessed 23 January 2024)]. To use D-MAINS, the code must be downloaded, copied, and pasted into the MATLAB platform. Adjusted_cell_count_nms.m is the cell segregation and cropping code, train_predict.m is the code for the convolutional neural network training, and prediction.m uses the neural network obtained from the previous code to classify the different cell statuses on non-training datasets.

Using the train_predict.m script in the GitHub folder, biologists can train the model with new cell types and datasets. The time it takes to train the model with a new dataset of dozens of thousands of images for different cell statuses and cell types varies depending on computing power and can range from 30 min to several hours using a standard laptop. Once trained, re-training is not necessary, and the information stored from the network will continually exist for future usage. Thus, different cell statuses and types can be trained using the same code. Using the prediction.m code file, the network saved and obtained from train_predict.m can be used to classify cells outside of the training; this process takes only seconds to minutes depending on the number of images. Therefore, D-MAINS can be used for real-time analysis. For both, adjusted_cell_count_nms_.m must be used to standardize the cropping so that each image is 100 × 100 pixels before use.

## 3. Results

### 3.1. Cell Segregation and Cropping

The success of D-MAINS relies on its ability to segregate accurately and crop cells in grayscale phase contrast conditions (without fluorescent labeling). As demonstrated in Figure 1h, this methodology proficiently captured a substantial proportion of objects within the microscopy images. The images underwent the cell cropping algorithm to gauge their precision, and a manual count was conducted to ascertain the actual number of cells present. The cell cropping algorithm exhibited an accuracy of 94.5% ± 1.5% across 3088 events. This accuracy aligns closely with the range observed in previous cell segregation methods, with the error rate typically falling between 4 and 7% [26]. While various new methods assert reliable whole-cell cropping, there are limitations in different imaging environments [27]. Conversely, the D-MAINS cell cropping method achieves a high accuracy, affirming the reliability and robustness of our conventional image processing approach. After testing different cell cropping sizes ranging from 70 × 70 pixels to 200 × 200 pixels, it was determined that 100 × 100 pixels was the most optimal for the high accuracy rate while maintaining a relatively low sample loss percentage (Figure 2). Within the process of cell cropping, errors manifested as false negatives and false positives. False negatives were observed when genuine objects went undetected, often because of their proximity to the edges of the image or when overlapping with other cell borders. Overlapping cells were judiciously identified and classified as “discarded cells” in accordance with the model’s training. Addressing edge-related detection issues could be achieved by capturing images with a larger field of view during acquisition. On the other hand, false positives were relatively infrequent, owing to a filtering mechanism that effectively screened out smaller debris. However, it is worth noting that a small fraction of the error—about 1%—stemmed from the presence of relatively large debris. This facet, although uncommon, contributed to the total margin of error within the process.

### 3.2. Successful Cell State Classification with D-MAINS

We first calculated the accuracy and sensitivity of D-MAINS using HeLa cells (a cervical cancer cell line), which were used for the training dataset. For HeLa cells, the accuracy was 96.4% ± 0.5%, and the sensitivities were 99.1% ± 0.3%, 98.3% ± 0.3%, 95.8 ± 0.8%, 96.5 ± 0.6%, and 99.8 ± 0.2% for mitosis, apoptosis, interphase, necrosis, and senescence, respectively (Figure 4). Note that multiplet cells, which are cells touching/overlapping each other, were discarded from calculations.

### 3.3. Verification of D-MAINS Classification Using Molecular Markers

**Figure 5:**
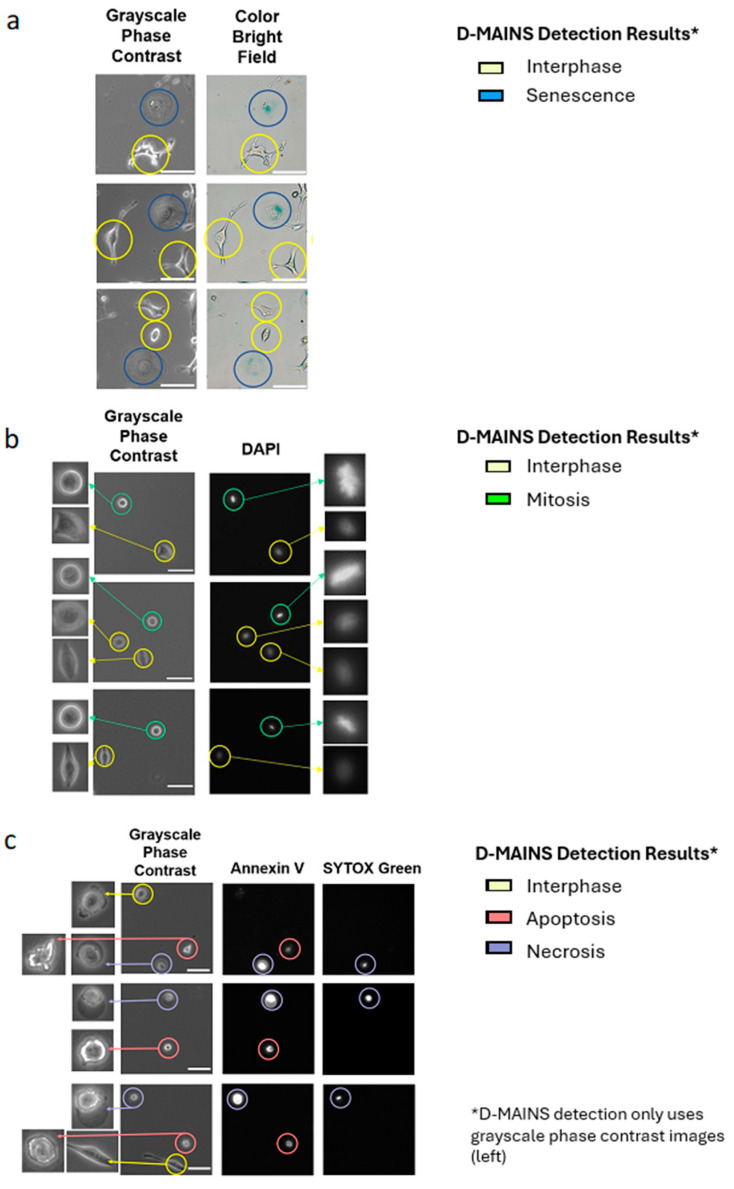
Validation of cell statuses with molecular markers. (**a**) Grayscale phase contrast images (left, used for label-free detection by D-MAINS) in comparison to β-galactosidase staining (blue) images shown in color bright field (right). Red represents “senescence” D-MAINS detection, and yellow represents the “interphase” D-MAINS detection. (**b**) A Hoechst 33342 stain was performed to stain the DNA in the cells. This stain is seen under the DAPI fluorescence channel (right). The grayscale phase contrast image (left) was used for D-MAINS detection. Purple circles represent “mitotic” D-MAINS detection and yellow circles represent “interphase” D-MAINS detection. Images on the left present the phase contrast images in a zoomed-in form to show morphology. Images on the right present the DAPI images in a zoomed-in form to show the condensed chromosome structure that confirms mitotic detection. (**c**) An Annexin V and SYTOX Green staining was performed to stain for apoptosis and necrosis. Annexin V stains for both apoptosis and necrosis, whereas SYTOX Green only stains for necrosis. This stain is seen under the Cy5 fluorescence channel for Annexin V and the GFP fluorescence channel for SYTOX Green. The phase contrast image (left) was used for D-MAINS analysis. Orange circles represent “apoptosis” D-MAINS detection, grey circles represent “necrosis” D-MAINS detection, and yellow circles represent “interphase” D-MAINS detection. Images on the left present the phase contrast images in a zoomed-in form to show morphology. Scale bar = 100 μm.

We also provided evidence of D-MAINS’s successful categorizations of apoptotic, necrotic, senescent, and mitotic cells correlated with their molecular biomarkers, consistently exceeding 90% accuracy. A β-galactosidase (β-gal) assay was conducted to validate D-MAINS’s precision in differentiating between senescent and interphase HeLa cells. This assay is specifically designed to stain senescent cells blue, a visually striking marker that is discernible under color bright field microscopy. As evidenced in Figure 5a, the cells were initially imaged under phase contrast microscopy (grayscale only), subjected to cropping and subsequent cell state categorization using D-MAINS, and then fixed for β-gal staining. Of the 34 detected “senescent” cells, 31 were stained as blue in the β-gal assay. The consistency between the detected “senescent” cells via D-MAINS using grayscale images and the same blue-stained cells (β-gal^+^) observed in the color bright field images affirms the algorithm’s precise detection of senescent cells. Furthermore, the absence of staining in cells identified as interphase corroborates the algorithm’s accurate identification of non-senescent cells.

In addition, a Hoechst staining protocol was employed to validate D-MAINS’s proficiency in distinguishing between mitotic and interphase cells [28]. As illustrated in Figure 5b, the cells were imaged under phase contrast microscopy (for D-MAINS analysis) as well as the DAPI fluorescence channel (for chromatin structure). We found cells categorized as “mitosis” by D-MAINS exhibited notably amplified and condensed chromosomes in contrast to the D-MAINS-categorized “interphase” cells. Of the 71 detected “mitosis” cells, 71 were stained by Hoechst, showing condensed chromatin. This validation substantiates the algorithm’s precision in accurately identifying mitotic cells.

Annexin V and SYTOX Green staining were also conducted to validate D-MAINS’s accuracy in distinguishing between apoptotic, necrotic, and living (interphase) cells. Apoptotic and necrotic cells should be positive for Annexin V, and only necrotic cells should be stained by SYTOX Green. Healthy interphase cells should not show any staining. As shown in Figure 5c, examining the side-by-side fluorescent staining and grayscale phase contrast imaging confirmed the successful detection of both “apoptosis” and “necrosis” by D-MAINS. Of the 65 detected “apoptosis” cells, 63 were stained by Annexin V but lacked Sytox Green. Additionally, of the 59 detected “necrosis” cells, 56 were stained by Annexin V and Sytox Green. This high accuracy rate validates the algorithm’s precision in identifying apoptotic and necrotic cells.

### 3.4. Validation of D-MAINS by Different Imaging Environments, Equipment, Magnifications and Cell Lines

**Figure 6:**
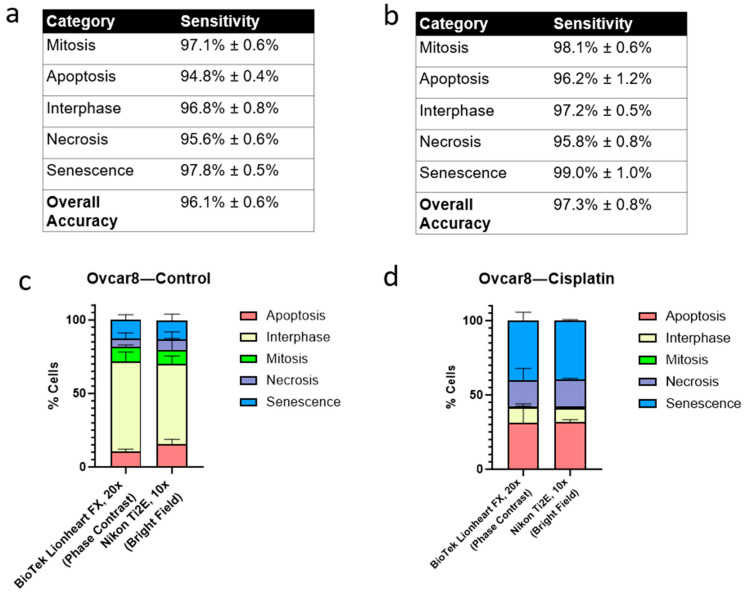
Assessment across different cell lines, treatments, microscopes, and magnifications using D-MAINS. (**a**) Table of accuracies and sensitivities of each cell status on Ovcar8 cells. (**b**) Table of accuracies and sensitivities of each cell status on NIH3T3 cells. (**c**,**d**) Control (**c**) and cisplatin-treated (**d**) Ovcar8 cells were imaged across two different microscopes and analyzed with the D-MAINS algorithm. BioTek Lionheart FX was the original microscope used for the training dataset and images at 20×. Nikon Ti2E was the other microscope used with 10× magnification. Note that Nikon Ti2E was not used to establish the D-MAINS training dataset. Phase contrast (BioTek Lionheart FX) and bright field (Nikon Ti2E) were used for imaging, respectively.

To test whether D-MAINS can function beyond the cell line that we used in the training dataset, we also employed the Ovcar8 cell line, a high-grade serous ovarian cancer cell line. Importantly, images of Ovcar8 cells were not included in the D-MAINS training dataset. We also used cisplatin to induce senescence in Ovcar8 cells, a method different from the senescence induction in HeLa cells (via etoposide) for D-MAINS training. The accuracy and specificity of D-MAINS on the Ovcar8 cell line were also conducted. The overall accuracy was 96.1% ± 0.6%, and the sensitivities were 97.1% ± 0.6%, 94.8% ± 0.4%, 96.8 ± 0.8%, 95.6 ± 0.6%, and 97.8 ± 0.5% for mitosis, apoptosis, interphase, necrosis, and senescence, respectively (Figure 6a), comparable to HeLa cells.

For even further validation, an immortalized murine fibroblast cell line (NIH3T3) was tested. This fibroblast cell line is fundamentally different from the previous cell lines that were epithelial. Images from NIH3T3 cells were not included in the D-MAINS training set and were only used for validation purposes. For induction of the different states of this cell line, the cells were treated with etoposide. The accuracy and specificity of D-MAINS on the NIH3T3 cell line were conducted. The overall accuracy was 97.3% ± 0.8%, and the sensitivities were 98.1% ± 0.6%, 96.2% ± 1.2%, 97.2% ± 0.5%, 95.8% ± 0.8%, and 99.0% ± 1.0% for mitosis, apoptosis, interphase, necrosis, and senescence, respectively (Figure 6b), comparable to HeLa cells.

To test D-MAINS’s flexibility, we also used two different microscopes with two objective lenses with different magnifications, the BioTek Lionheart FX 20× magnification (phase contrast) and the Nikon Ti2E 10× magnification (bright field), respectively, for image capture of the cells under the same treatments. Note that to standardize the images for image processing purposes, the 10× magnification images were zoomed in to simulate 20× magnification using MATLAB. As shown in Figure 6c,d, the cisplatin-induced Ovcar8 cells showed a significant amount of senescence and cell death, which is expected; additionally, in the control groups, where no treatment was added, most cells were in the interphase state. The two different microscopes had similar results, showing that the type of microscope used did not greatly affect the D-MAINS classification. Thus, Ovcar8 cells should be considered as one successful model showing that D-MAINS was able to accurately categorize different cell statuses across a different cell line and microscopes (Figure 6c,d).

## 4. Discussion

In this work, we developed D-MAINS, an algorithm using a convolutional neural network that offers a label-free, high-throughput method for quantitatively analyzing different cell statuses within adherent cancer cell lines. Additionally, there is also potential for future works to use D-MAINS for real-time or continuous imaging, which is beneficial for tracking the growth and kinetics of biological experiments. The D-MAINS model also shows high flexibility and is able to assess cell statuses across different cell lines, microscopes, magnifications, and bright field/phase contrast grayscale images.

With D-MAINS, we achieved relatively high accuracy rates of 96.4 ± 0.5% (HeLa), 96.1% ± 0.6% (Ovcar8), and 97.3% ± 0.8% (NIH3T3). Our experiments substantiate predictions with remarkable precision, consistently exceeding 90% accuracy when verified with molecular biomarkers. This means that most cases are correctly assessed with the algorithm. We also carefully examined the wrongly classified cases. A specific case that was carefully examined was the distinction between apoptotic and necrotic cells; some necrosis cells were improperly categorized as apoptosis. This is likely due to the original microscopy image not clearly showing the dark cell swelling. Thus, it is important that the microscopy images must have sufficient contrast and brightness in order to show the entire cell clearly. However, this does not mean that the contrast and the brightness of the images have to be the same for D-MAINS to categorize different cell states, given that we tested two different imaging setups on the BioTek Lionheart FX and the Nikon Ti2E and saw similar results (Figure 6b,c).

Another specifically examined case involved distinguishing between early-stage senescence and interphase cells. Early-stage senescence is characterized by chromatin remodeling, which is not immediately evident morphologically (flat and dark) [29,30]. Because senescent cells generally become more flattened as they become more senescent, early-stage senescent cells are of comparable size and morphology to interphase cells. Because of this feature, it is also important to note that the model can only distinguish between different cell statuses that can be easily distinguished morphologically. If the cells seem to be in either cell state at the early stage, the model will also have a hard time distinguishing between the two. Considering that morphology changes may happen earlier or later than certain molecular biomarkers during either cell death or the senescence process, some cases of D-MAINS classification may not always align with those biomarkers. However, even when using biomarkers, markers that can 100% classify a senescent cell are also not definitive yet. Because of the nature of senescence, we acknowledge that the detection of senescent cells could be tricky, but we believe our morphological analysis via D-MAINS will add one more standard to help better probe senescence. Since the β-gal assay is compatible with D-MAINS detection, as shown in Figure 5, D-MAINS together with the β-gal assay can better determine senescent cells compared with the β-gal assay alone. Furthermore, D-MAINS is simple, label-free, and can be performed in real time. To our knowledge, no such method for senescent cell probing is available so far. Thus, we believe our work represents an advance to our community.

It is also important to note that our training dataset only includes HeLa cells, so D-MAINS would only be accurate to those morphologically similar to HeLa cells and are adherent cell lines. In addition, the D-MAINS algorithm also relies on cropped single-cell images. If the cells are too confluent, then many images with multiple cells would be discarded, even with a small 100 × 100-pixel cropping size. Thus, a low confluence cell culture condition was used. The algorithm will still work with higher confluent cells. However, more cells will be discarded, which may lead to biased counting. To improve this model, the ultimate solution would be to significantly increase the cell diversity and quantity in the training dataset, which is out of the capacity of a normal-sized research lab in academia. However, this work provides the first “proof-in-principle” example that a deep learning-based AI model can probe various cell statuses solely based on their morphology in grayscale phase contrast and bright field images. Our work confirms that label-free cell status classification is feasible and accountable.

## Data Availability

The D-MAINS algorithm was created in MATLAB R2023a, and the code was uploaded to GitHub as a public source code [https://github.com/cakekio/D-MAINS (accessed 23 January 2024)].
